# Mechanisms of cytokine release syndrome and neurotoxicity of CAR T-cell therapy and associated prevention and management strategies

**DOI:** 10.1186/s13046-021-02148-6

**Published:** 2021-11-18

**Authors:** Xinyi Xiao, Shengkang Huang, Sifei Chen, Yazhuo Wang, Qihang Sun, Xinjie Xu, Yuhua Li

**Affiliations:** 1 The Second School of Clinical Medicine, Zhujiang Hospital, Southern Medical University, Guangzhou, 510282 People’s Republic of China; 2 Medical College of Rehabilitation, Southern Medical University, Guangzhou, Guangdong 510515 People’s Republic of China; 3 State Key Laboratory of Ophthalmology, Zhongshan Ophthalmic Center, Sun Yat-sen University, Guangzhou, Guangdong 510623 People’s Republic of China; 4 State Key Laboratory of Cardiovascular Disease, Fuwai Hospital, National Center for Cardiovascular Diseases, Chinese Academy of Medical Sciences and Peking Union Medical College, Beijing, 100037 People’s Republic of China; 5 Department of Hematology, Zhujiang Hospital, Southern Medical University, Guangzhou, 510282 People’s Republic of China; 6 Bioland Laboratory (Guangzhou Regenerative Medicine and Health Guangdong Laboratory), Guangzhou, Guangdong 510005 People’s Republic of China

**Keywords:** CAR T-cell, Cytokine release syndrome (CRS), Immune effector cell-associated neurotoxicity syndrome (ICANS), Neurotoxicity, Mechanisms, Management, Strategies

## Abstract

Chimeric antigen receptor (CAR) T-cell therapy has yielded impressive outcomes and transformed treatment algorithms for hematological malignancies. To date, five CAR T-cell products have been approved by the US Food and Drug Administration (FDA). Nevertheless, some significant toxicities pose great challenges to the development of CAR T-cell therapy, most notably cytokine release syndrome (CRS) and immune effector cell-associated neurotoxicity syndrome (ICANS). Understanding the mechanisms underlying these toxicities and establishing prevention and treatment strategies are important. In this review, we summarize the mechanisms underlying CRS and ICANS and provide potential treatment and prevention strategies.

## Background

Chimeric antigen receptor (CAR) T-cell therapy, one of the most significant developments in immunotherapy, has yielded impressive outcomes in hematological malignancies. To date, five different CAR T-cell products have been approved by the US Food and Drug Administration (FDA), including four products targeting CD19 for acute lymphocyte leukemia (ALL) or lymphoma [1–4] and idecabtagene vicleucel (Abecma) targeting B cell maturation antigen (BCMA), which has recently been approved for relapsed or refractory multiple myeloma (R/R MM) [5].

Genetically engineered to express CAR molecules that can specifically recognize tumor antigens, CAR T-cells can be activated, proliferate and exert antitumor effects without major histocompatibility complex (MHC) restriction. With the optimization of products and treatment regimens, the efficacy of CAR T-cell therapy is improving, and its application fields are expanding. Despite these achievements, some severe toxicities associated with CAR T-cells dampen their development. The most common toxicity is cytokine release syndrome (CRS), which is a systemic inflammatory response mediated by the overactivation of effector cells and large amounts of cytokines [6]. Neurotoxicity, another common toxicity related to CAR T-cell therapy, is a toxic encephalopathy state with a broad spectrum of neuropsychiatric symptoms. Such neurotoxicity has been designated “immune effector cell-associated neurotoxicity syndrome (ICANS)” by the American Society for Transplantation and Cellular Therapy (ASTCT) [7]. Clarifying the mechanisms underlying CRS and ICANS could facilitate the prevention and treatment of CAR T-cell-related toxicities. Herein, we review the key pathways involved in the mechanisms of CRS and ICANS based on the current understanding and provide promising prevention and management strategies to improve the safety of this beneficial therapy and expand its application. Since CAR T-cell-related toxicities are investigated mostly in the field of hematological malignances, they are the focus of this review.

## Current understanding of the mechanisms of CRS

**Fig. 1:**
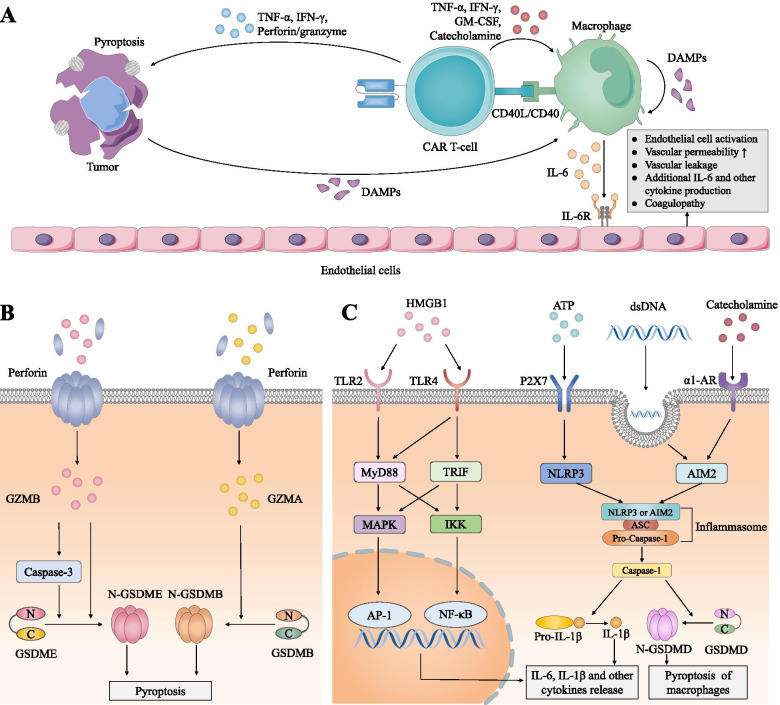
Mechanisms of CRS. **A.** Cell interactions involved in CRS. Upon recognizing tumor antigens, CAR T-cells secrete perforin, granzyme and inflammatory cytokines, including IFN-γ and TNF-α, to induce pyroptosis of tumor cells, releasing large amounts of DAMPs that stimulate macrophages for massive cytokine production and CRS. Macrophages can also be activated by cytokines, such as GM-CSF, IFN-γ, TNF-α and catecholamine, or CD40/CD40L interactions with CAR T-cells. Pyroptosis of macrophages and further DAMPs leakage amplify the inflammatory cascade. IL-6 and other cytokines in CRS bind to their receptors on endothelial cells, causing an increase in vascular permeability and leakage and promoting cytokine production to exacerbate the CRS. **B.** Signaling pathway of pyroptosis in tumor cells. GZMA or GZMB enters tumor cells through perforin-induced pores. GZMB cleaves GSDME or activates caspase-3 to cleave GSDME. GZMA cleaves and activates GSDMB. Subsequently, the released gasdermin-N domain (N-GSDME or N-GSDMB) oligomerizes on the cell membrane to form membrane pores and disrupts the osmotic potential, resulting in cell swelling and lysis. **C.** Inflammatory signaling pathways in macrophages. Pyroptotic products include HMGB1, ATP, and dsDNA. HMGB1 activates TLR2 and TLR4 and subsequently recruits MyD88 and TRIF to activate MAPKs and IKK, leading to the subsequent production of inflammatory cytokines via AP-1 and NF-κB; ATP binds to the P2X7 receptor and induces NLRP3 activation; dsDNA is phagocytized by macrophages and activates AIM2. Activated AIM2 or NLRP3 combines with ASC and pro-caspase-1 to induce the maturation of caspase-1, which can cleave the N-terminus of GSDMD to form pores on the membrane, triggering pyroptosis and producing bioactive IL-1β. In addition, catecholamine can be recognized by α1-AR and activate the AIM2/ASC-caspase-1 pathway

CRS is the most common toxicity related to CAR T-cell therapy, with an incidence of 42–100%, and 0–46% of patients develop severe CRS after CAR T-cell infusion (Table 1). It is believed that CRS is a systemic disease induced by the overactivation of immune effector cells and supraphysiological levels of various proinflammatory cytokines, including IL-1, IL-6, IFN-γ, and granulocyte-macrophage colony-stimulating factor (GM-CSF) [7]. This toxicity manifests as a constellation of symptoms (Table 2**)**, most of which are reversible; 0–9.1% of patients progressed to fatal cases (Table 1), whereas there were no deaths from CRS in most clinical trials. A meta-analysis of 2592 patients from 84 eligible studies showed that the mortality rate of CRS was less than 1% [8]. A schematic representation of the CRS mechanisms is briefly shown in Fig. 1A. The interplay between CAR T-cells and tumor cells activates host bystander cells, especially macrophages, eliciting a distortion of the cytokine network. Then, massive cytokines induce endothelial cell activation, contributing to constitutional symptoms in relation to CRS.

**Table 1:** Selected published clinical trials of CAR T-cell therapy

Trial	N	Target	Costimu-latory domain	CR (%)	Overall survival rate	CRS and ICANS grading criteria	CRS (%)	Severe CRS* (%)	ICANS (%)	Severe ICANS* (%)	Toxicity related mortality	Refs
**ALL**												
Maude et al. 2014	30	CD19	4-1BB	27(90)	78% (6 m)	CTCAE†	30(100)	8(27)	13(43)	NR	0	[31]
Lee et al. 2015	20	CD19	CD28	14(70)	50% (12 m)	CTCAE†	16(80)	6(30)	6(30)	1(5)	0	[30]
Turtle et al. 2016	30	CD19	4-1BB	27(93)	NR	CTCAE†	25(83)	7(23)	15(50)	15(50)	1 CRS 1 ICANS	[84]
Gardner et al. 2017	43	CD19	4-1BB	40(93)	69.5% (12 m)	CTCAE†	40(93)	10(23)	21(49)	9(21)	0	[205]
Maude et al. 2018	75	CD19	4-1BB	61(81)	76% (12 m)	PENN/CHOP CTCAE	58(77)^P^	35(46)	30(40)^C^	10(13)	1 ICANS	[206]
Park et al. 2018	53	CD19	CD28	44(83)	50% (12.9 m)	MSKCC CTCAE	45(85)^M^	14(26)	23(44)^C^	22(42)	1 CRS	[117]
Frey et al. 2020	35	CD19	4-1BB	24(69)	50% (19.1 m)	PENN/CHOP CTCAE	33(94)^P^	6(17)	14(40)^C^	2(6)	3 CRS	[109]
Fry et al. 2018	21	CD22	4-1BB	12(57)	NR	CTCAE†	16(76)	0(0)	6(28)	0(0)	0	[80]
Shah et al. 2020	58	CD22	4-1BB	40(70)	50% (13.4 m)	Lee CTCAE ASTCT	50(86)^L^	5(10)^L^ 12(24)^A^	19(33)^C^	1(2)	0	[81]
**NHL**												
Turtle et al. 2016	32	CD19	4-1BB	11(34)	‡	CTCAE†	20(63)	4(13)	9(28)	9(28)	1 ICANS	[85]
Schuster et al. 2017	28	CD19	4-1BB	16(57)	57% (28.6 m)§	PENN/CHOP CTCAE	16(57)^P^	5(18)	11(39)^C^	3(11)	1 ICANS	[87]
Neelapu et al. 2017	101	CD19	CD28	55(54)	52% (18 m)	Lee CTCAE	94(93)^L^	13(13)	65(64)^C^	28(28)	2 CRS	[89]
Schuster et al. 2019	111	CD19	4-1BB	37(40)	50% (12 m)	PENN/CHOP CTCAE	64(58)^P^	24(22)	23(21)^C^	13(12)	0	[118]
Abramson et al. 2020	269	CD19	4-1BB	136(53)	58% (12 m)	Lee CTCAE	113(42)^L^	6(2)	80(30)^C^	27(10)	0	[207]
**MCL**												
Wang et al. 2020	68	CD19	CD28	40(67)	83% (12 m)	Lee CTCAE	62(91)^L^	10(15)	43(63)^C^	21(31)	0	[208]
**MM**												
Brudno et al. 2018	16	BCMA	CD28	10(63)	50% (7.1 m)¶	Lee	15(94)^L^	6(38)	NR	NR	NR	[209]
Zhao et al. 2018	57	BCMA	4-1BB	39(68)	50% (15 m)§	Lee CTCAE	51(90)^L^	4(7)	1(2)^C^	0(0)	NR	[210]
Cohen et al. 2019	25	BCMA	4-1BB	2(8)	50% (502d)	PENN/CHOP CTCAE	22(88)^P^	8(32)	8(32)^C^	3(12)	0	[211]
Raje et al. 2019	33	BCMA	4-1BB	15(45)	50% (11.8 m)§	Lee CTCAE	25(76)^L^	2(6)	14(42)^C^	1(3)	0	[212]
Munshi et al. 2021	128	BCMA	4-1BB	42(33)	78% (12 m)	Lee CTCAE	107(84)^L^	7(5)	23(18)^C^	4(3)	1 CRS	[213]
**CLL**												
Porter et al. 2015	14	CD19	4-1BB	4(29)	50% (29 m)	CTCAE†	9(64)	6(43)	6(43)	1(7)	0	[214]
Turtle et al. 2017	24	CD19	4-1BB	4(21)	50% (6.6 m)	Lee CTCAE†	20(83)^L^	2(8)	8(33)^C†^	6(25)	1 CRS and ICANS	[86]
Frey et al. 2020	38	CD19	4-1BB	9(28)	50% (64 m)	PENN/CHOP CTCAE ASTCT	24(63)^P^ 23(59)^A^	9(24) 4(11)	3(8)^C^	0(0)	0	[215]

*CAR* Chimeric antigen receptor, *N *number of patients, *CR* Complete remission, *CRS* Cytokine release syndrome, *ICANS* Immune effector cell-associated neurotoxicity syndrome, *Refs* References, *ALL* Acute lymphoblastic leukemia, *NHL* Non-Hodgkin lymphoma, *MCL* Mantle cell lymphoma, *MM* Multiple myeloma, *CLL* Chronic lymphocytic leukemia, *NR* No report, *m* Months, *d* Days, *h* Hours, *Cy* Cyclophosphamide, *Flu* Fludarabine, *E* Etoposide

CRS and ICANS grading systems used by each trial are denoted by superscripts as follows: *A* ASTCT criteria, *C* CTCAE criteria, *L* Lee criteria, *P* PENN/CHOP criteria, *M* MSKCC criteria

* Severe CRS/ICANS are defined as CRS/ICANS ≥ Grade 3

† Modified criteria

‡ 25 m 50% vs 6.3 m 50% (Cy/Flu Group vs Cy or Cy/E Group)

§ Progression-free survival

¶ Event-free survival

**Table 2:** Summary of the clinical features of CRS and ICANS

	CRS	ICANS
**Symptoms & Signs**	**Onset:** Fever with other constitutional symptoms (myalgias, malaise, nausea, vomiting, diarrhea, etc.) **Progression:** Hypotension, hypoxia, tachycardia, tachypnea, arrhythmia, pleural effusion, capillary leak, coagulopathy, pulmonary edema, DIC and multiorgan failure [7, 155, 216] **Accompanied infections** [217] **L-CRS (in NHL):** Local swelling and redness [43]	**Onset:** Somnolence, disorientation, inattention, tremor, expressive aphasia, dysgraphia and apraxia [84, 218] **Progression:** Globe aphasia, cognitive disturbance, focal motor and sensory defects, seizures, fatal cerebral edema and intracranial hemorrhage [155, 219] **Long-term sequelae** [66, 220]
**Timing**	**Onset:** 1–9 days after CAR T-cells infusion **Duration:** 5–11 days [81, 89, 118, 206–208, 210, 211, 213]	**Onset:** 2–9 days after CAR T-cells infusion **Duration:** 3–17 days [89, 118, 207, 208, 213]
**Cytokine profile**	IL-6, IFN-γ, TNF-α, GM-CSF, IL-10, MIP-1, MCP-1 [44, 221]	**Serum:** IFN-γ, IL-15, IL-6, IL-10, GM-CSF, IL-1RA, IL-2, IP-10 IL-1β, IL-8, and TNF **CSF:** Similar to the cytokine profile in the serum, except for higher levels of IL-8, IP-10, and MCP-1 [45, 222]
**Risk factors**	**Patient Characters:** Disease type (ALL), high disease burden, preexisting thrombocytopenia and endothelial activation **Characters of CAR T-cell products:** Targeting CD19, CD28 costimulatory domain, receiving fludarabine and cyclophosphamide, high infusion dose, peak serum CAR T-cells levels [146, 223]	**Patient Characters:** CRS, disease type (ALL), high disease burden, preexisting thrombocytopenia and endothelial activation, preexisting neurologic comorbidities **Characters of CAR T-cell products:** Targeting CD19, CD28 costimulatory domain, receiving fludarabine and cyclophosphamide, high infusion dose, peak serum CAR T-cells levels [146, 223]
Grading criteria*	•Temperature ≥ 38.0 °C •Hypotension (based on vasopressor) •Hypoxia	•ICE score (for adults and children> 12 years) or CAPD (for children≤12 years) •Depressed level of consciousness •Seizures •Motor findings •Elevated intracranial pressure/cerebral edema
**Management**	•Antipyretics, IV hydration, anti-infective treatment •Tocilizumab, corticosteroids •ICU treatment, vasopressor support, supplemental O_2_ [224] •Symptomatic treatment for L-CRS (e.g. drainage of serous effusion, airway protection, regulation of intestinal flora) [43]	•Supportive management •EEG, neuroimaging •Tocilizumab (only when concurrent with CRS), corticosteroids, anti-epileptics drugs •ICU treatment, airway protection, specific neurointensive treatment [224]

*CRS* Cytokine release syndrome, *ICANS* Immune effector cell associated neurotoxicity syndrome, *DIC* Disseminated intravascular coagulation, *L-CRS* Local-Cytokine release syndrome, *NHL* Non-Hodgkin’s lymphoma, *CAR* Chimeric antigen receptor, *IL* Interleukin, *IFN-γ* Interferon-γ, *TNF-α* Tumor necrosis factor-α, *GM-CSF* Granulocyte-macrophage colony-stimulating factor, *MIP* Macrophage inflammatory protein, *MCP* Monocyte chemoattractant protein, *ALL* Acute lymphoblastic leukemia, *IL-1RA* Interleukin-1 receptor agonist, *IP-10* Interferon-γ-inducible protein 10, *ICE* Immune effector cell–associated encephalopathy, *CAPD* Cornell Assessment of Pediatric Delirium, *IV* intravenous, *ICU* Intensive care unit, *EEG* Electroencephalogram

* Based on the ASTCT consensus, which is applicable to systemic CRS and ICANS. A grading criteria for L-CRS has recently been proposed [43]

### CAR T-cell activation and pyroptotic target cells: the root factor

After the recognition of tumor antigens, CAR T-cells release massive amounts of perforin/granzymes and cytokines, including TNF-α and IFN-γ, resulting in tumor pyroptosis [9–12]. Pyroptosis is a type of programmed cell death that differs from apoptosis [13], and is characterized by cellular swelling, lysis and subsequent cell content and proinflammatory factor release. It is believed that pyroptosis of the target cell represents the onset of CRS. Two pathways are likely to be involved, which are mediated by granzyme B (GZMB) and granzyme A (GZMA) (Fig. 1B). GZMA and GZMB can both enter cells through pores formed by perforin [9, 12]. Subsequently, GZMB cleaves gasdermin E (GSDME) directly or by activating caspase-3 [9], while GZMA directly cleaves gasdermin B (GSDMB) for its activated form [12]. Then, the N-domains of gasdermin, which are veiled by the C-terminus, can be released and oligomerize on the cell membranes to form pores, causing decreased cell viability, bubbles blowing from the plasma membrane, cell swelling and finally cell lysis [9, 13, 14]. The different types of gasdermin and their pyroptotic pathways differ among tumor cells. GSDME widely exists in hematologic malignances [9], while GSDMB is found more frequently in bladder cancer, skin cancer and renal clear cell carcinoma, and its expression can be upregulated by cytokines, such as IFN-γ [12].

Cell death through either apoptosis or pyroptosis mainly depends on the amount of gasdermin expression [9, 12, 15]. Low levels of gasdermin induce apoptosis, while high levels of gasdermin switch apoptosis to pyroptosis [9]. Cytotoxic T lymphocytes (CTLs) mediate apoptosis in tumor cells via a low level of perforin/granzyme release, consequently activating little gasdermin and producing a few pores on the cytoplasmic membrane [16, 17]. Notably, cells are capable of repairing perforin pore formation to a certain degree, protecting cells from pyroptosis [18]. In contrast, CAR T-cells release a large amount of perforin/granzymes to induce massive gasdermin release, surpassing their self-repair capability and leading to pyroptosis [9]. Clinically, GSDME is widely expressed in B leukemic cells after CD19 CAR T-cell infusion, and the severity of CRS is positively associated with an increase in GSDME [9].

### Activated macrophages: the key mediator

An increasing number of studies indicate that monocyte and macrophage lineages are the key origin of inflammatory cytokines in relation to CRS [19–21]. Macrophages can be activated by damage-associated molecular patterns (DAMPs), including high-mobility group box 1 (HMGB1), adenosine 5′-triphosphate (ATP) and double-stranded DNA (dsDNA) [9, 22], which are pyroptotic products of tumor cells (Fig. 1C). HMGB1 can bind Toll-like receptor 2 (TLR2) and TLR4 on the surface of macrophages. Then, the adaptor proteins myeloid differentiation primary-response 88 (MyD88) and TIR-domain-containing adaptor inducing IFNβ (TRIF) recruit and activate mitogen-activated protein kinases (MAPKs) and IκB kinase (IKK). MAPKs and IKK regulate the release of a wide range of cytokines, including IL-6, via transcription factors activator protein 1 (AP-1) and nuclear factor κB (NF-κB) [9, 22]. In addition, TLR2 induces soluble IL-6R (sIL-6R) secretion, which can combine with IL-6 and facilitate the proinflammatory effects of IL-6 [23]. ATP, which is recognized by its receptor P2X7 on macrophages, can induce the activation of NOD-, LRR- and pyrin domain-containing 3 (NLRP3) in the cytoplasm and recruit apoptosis-associated speck-like protein (ASC) and pro-caspase 1 to form the NLRP3 inflammasome, subsequently leading to the maturation of caspase 1 [22]. On the one hand, caspase 1 is responsible for pro-IL-1β cleavage and the secretion of IL-1β; on the other hand, caspase 1 transforms GSDMD into the active form and leads to pyroptosis in macrophages [9, 22]. Pyroptotic macrophages consequently produce more DAMPs and proinflammatory factors, creating a vicious cycle and leading to the further activation of macrophages [22]. Additionally, the dsDNA-mediated absent in melanoma 2 (AIM2) inflammasome pathway could be involved in caspase 1 formation [24]. Macrophages phagocytize dsDNA released by pyroptotic tumor cells and activate the AIM2 inflammasome, which is a dsDNA sensor, in the cytoplasm. Similar to the NLRP3 inflammasome pathway, activated AIM2 forms an AIM2/ASC-pro-caspase 1 complex to trigger caspase 1-dependent IL-1β maturation [24], thereby causing more IL-1β release and aggravation of CRS.

In addition to the “pyroptosis–DAMPs–macrophage” pattern, macrophages can be recruited and activated by cytokines produced by CAR T-cells, such as TNF-α, IL-2, GM-CSF and IFN-γ [25, 26]. Contact-dependent CD40 ligand (CD40L)-CD40 interactions between CAR T-cells and macrophages may also play a role in triggering IL-6 and IL-1 release and higher inducible nitric oxide synthase (iNOS) expression [21]. iNOS stimulates NO production, resulting in potentiated vasodilatation and hemodynamic instability, which are common clinical features of CRS [27]. In addition, catecholamines are produced after the coculture of CAR T-cells and malignant cells, which may be involved in cytokine release [24, 28]. By binding α1-adrenergic receptors (ARs) on the surface of macrophages, catecholamines can enhance the AIM2/ASC-caspase-1 pathway and further promote IL-1β production and macrophage pyroptosis [22, 24]. In addition, catecholamines lead to a self-amplifying feed-forward loop in macrophages, promoting the release of catecholamines, IL-6 and other cytokines, including macrophage inflammatory protein (MIP)-1α, IFN-γ, IL-2 and TNF [28]. Monocytes are also likely to be the key mediators and the main source of IL-6 and IL-1 in CRS, as Norelli et al. reported that monocyte depletion prior to CAR T-cell therapy can protect mice from lethal CRS [20].

### IL-6 and endothelial cell activation: the final core pathway

Among the cytokines released by monocytes/macrophages, including IL-6, IL-1, IL-2, TNF-α, GM-CSF and IFN-γ, IL-6 plays a central role [29–31]. Notably, in addition to macrophages and monocytes, dendritic cells, endothelial cells, and even CAR T-cells are considered to participate in IL-6 production [21, 32]. IL-6 activates its downstream Janus kinase (JAK) and signal transducer and activator of transcription 3 (STAT3) mainly by binding the membrane-bound IL-6 receptor (mIL-6R) (classic signaling) or sIL-6R (trans-signaling) and another membrane protein, gp130. gp130 is ubiquitously expressed, whereas mIL-6R is mainly expressed on hepatocytes and immune cells. Cells that do not express mIL-6R, such as endothelial cells, are activated by trans-signaling in which IL-6 binds sIL-6R and forms a complex in the serum, triggering the dimerization of gp130 on the cell membrane [33–35]. As a result, activated endothelial cells secrete additional IL-6 and other proinflammatory factors, such as vascular endothelial growth factor (VEGF), IL-8, monocyte chemoattractant protein-1 (MCP-1) and coagulation cascade activator plasminogen activator inhibitor-1 (PAI-1) [36–38], leading to a positive loop of cytokine release and amplified inflammatory responses. In addition, endothelial cells are injured by cytokines and contribute to increased vascular permeability and leakage, edema, organ hypoperfusion, coagulopathy and organ dysfunction [39]. In CRS-associated coagulopathy, tissue factor (TF), factor VIIa, factor Xa, thrombin and platelets can also play roles as proinflammatory factors, upregulating cytokine synthesis in endothelial cells [40–42].

In addition to the most common systemic CRS, it is reported that local CRS (L-CRS) is the earliest toxicity in non-Hodgkin lymphoma (NHL) and mainly manifests as local swelling and redness [43]. Initially, CAR T-cells distribute locally in the tumor mass, triggering L-CRS. Then, with CAR T-cell and cytokine accumulation, they overflow into the circulation and cause systemic CRS [43]. However, the mechanisms of L-CRS are likely to be unique since the inhibition of IL-6 may aggravate L-CRS. More research investigating its specific mechanisms is warranted.

## Current understanding of the mechanisms of ICANS

**Fig. 2:**
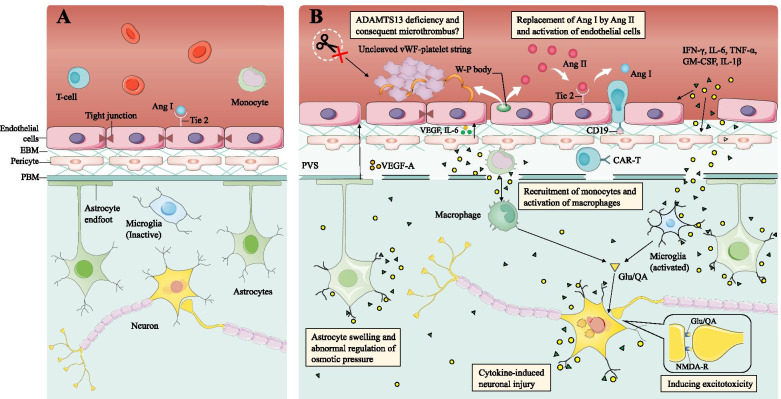
Mechanisms of ICANS. **A.** Normal state. BBB is integral, consisting of endothelial cells with tight junctions, EBM, pericytes, PBM and endfeet of astrocytes. Tie2 on endothelial cells binds with Ang I to maintain the quiescent state of endothelium. **B.** ICANS. Systemically increased cytokines, such as IFN-γ, IL-6, GM-CSF and TNF, can activate brain endothelial cells to release W-P bodies and their contents, Ang II and vWF. Platelets adhere vWF to form the vWF-platelet string. Possibly because of the lack of ADAMTS13, vWF fails to be cleaved and thus causes microvascular thrombosis and consumptive coagulopathy. An increase in the Ang II/Ang I ratio can lead to endothelial activation and BBB disruption through abnormalities of the Ang-Tie2 axis. As a result, cytokines and CAR T-cells infiltrate the peripheral vascular space (PVS). Cytokines have access to pericytes, inducing pericyte stress and consequent VEGF and IL-6 release to further activate endothelial cells. CD19 CAR T-cells trigger CD19-positive pericyte depletion. Astrocytes can also be injured by cytokines, causing cell swelling, abnormal osmotic forces, and consequently cerebral edema. In addition, when stimulated by cytokines, astrocytes produce VEGF-A to aggravate the BBB disruption. The disrupted BBB allows myeloid cells to infiltrate into the brain parenchyma, cooperating with activated resident microglia to trigger the immune response in the CNS. Macrophages and microglia can also produce QA and Glu, activating NMDA receptors on synapses to induce seizures and other excitotoxicity. Cytokines may also play a role in neuronal injury

ICANS is the second most common toxicity associated with CAR T-cell therapy, with incidences ranging from 2% to 64% for all-grade ICANS and 0–50% for severe ICANS (Table 1). ICANS is strongly related to CRS and always occurs and reaches a peak several days after CRS [39, 44–47] despite some rarely independent occurrences [39, 48]. The clinical presentations are summarized in Table 2. The brain blood barrier (BBB) plays an important role in maintaining homeostasis of the central nervous system (CNS). In an inflammatory state, the integrity of the BBB is disrupted, triggering a series of pathophysiological reactions in the brain. It appears that the activation of the cerebral vascular endothelium and disruption of the BBB occur at the onset of ICANS. Exposure of other components of the BBB, especially astrocytes and pericytes, to large amounts of cytokines is likely to cause injury, cytokine secretion and further breakdown of the BBB. Without the barrier function of the BBB, extensive numbers of immune cells and cytokines infiltrate the CNS. Together with activated resident proinflammatory cells, infiltrated immune cells exacerbate the inflammatory cascade in the CNS, accounting for the cerebral edema, thrombosis, hemolysis and other neuropsychiatric symptoms observed in ICANS (Fig. 2).

### Brain vascular endothelial activation and BBB disruption

The BBB consists of endothelial cells with tight junctions, the endothelial basement membrane (EBM), pericytes surrounding capillaries, the parenchymal basement membrane (PBM) and the endfeet of astrocytes, also called glia limitans [49, 50]; of these components, the endothelium is the most important component. Some characteristics of brain vascular endothelial cells are distinct from those of peripheral vascular endothelial cells, such as tight junctions and specific transport systems, restricting various molecules and cells from entering the CNS [51, 52]. However, inflammation can lead to endothelial activation, reversible contraction, damage to tight junctions, and even cell death [53, 54], disrupting the integrity of the BBB and activating coagulation. The angiopoietin (Ang)-Tie2 axis plays a significant role in balancing the endothelium between quiescence and activation. Ang I is produced by platelets and pericytes constitutively, while Ang II is primarily reserved in Weibel-Palade (W-P) bodies in endothelial cells. Normally, Ang I binds Tie2 on endothelial cells, promoting cell spreading and accumulating vascular endothelial cadherin (VE cadherin), which is an important component of tight junctions. This characteristic ensures the integrity of the endothelial barrier [55, 56]. When stimulated by inflammatory factors, such as VEGF, thrombin and epinephrine, W-P bodies and Ang II are released from the endothelium via Ca^2+^-mediated or cAMP-mediated pathways [57]. As a result, concentrated Ang II in serum replaces Ang I, inhibiting the Tie2 pathway and increasing the permeability of the endothelium [58, 59]. Disorder of the Ang-Tie2 axis is likely to be implicated in CAR T-cell-associated ICANS, and severe ICANS patients have been observed to present a higher ratio of Ang II/Ang I [39, 45]. Interestingly, another study showed that a decrease in Ang I triggered a higher Ang II/Ang I ratio rather than increased Ang II, which is associated with the thrombocytopenia observed in severe ICANS patients [45].

Endothelial activation is likely to contribute to ICANS-associated microthrombi and disseminated intravascular coagulation (DIC). Laboratory markers indicate that patients with severe ICANS suffer DIC, and an autopsy study showed intravascular von Willebrand factor (vWF) binding and platelet microthrombi [39, 45]. vWF can be released from W-P bodies in the form of ultralarge vWF and bind endothelial cells [60]. Then, vWF exposes the binding sites for platelet adhesion and vWF-platelet string formation. The blood flow shearing force induces vWF to expose another binding site for a disintegrin and metalloproteinase with thrombospondin type 1 repeats member 13 (ADAMTS13), which is responsible for vWF cleavage [60, 61]. Cytokines influence this process. IFN-γ, TNF, IL-4 and IL-6 can directly inhibit the production of ADAMTS13 or impede its cleavage [61, 62]. It is assumed that thrombocytopenia and consumptive coagulopathy in severe ICANS patients are due to ADAMTS13 deficiency [39], which is similar to the mechanism responsible for thrombotic thrombocytopenic purpura (TTP) [63]. However, since there is no evidence of other systematic manifestations of thrombotic microangiopathy, such as renal failure and morphological changes in red blood cells, thrombotic microangiopathy in ICANS presents a unique pathogenesis requiring further investigation.

### Dysfunction of other components of the BBB and inflammation amplification

Detrimental cytokines could be accessible to other components of the BBB due to the high permeability of the endothelial barrier, exacerbating the inflammatory response and further damaging the integrity of the BBB. Astrocytes represent a component with endfeet in the inner layer of the BBB and have direct contact with the brain parenchyma, regulating the osmotic pressure of the brain [64, 65]. Autopsy studies of severe ICANS patients have revealed that astrocytes are activated and injured [39, 66, 67], and the astrocyte markers glial fibrillary acidic protein (GFAP) and calcium-binding protein B (S100b) [68, 69] are increased in the CSF of ICANS patients after treatment with CD19 CAR T-cells [66]. Astrocyte injury is likely to play a role in the development of CAR T-cell-associated cerebral edema [66]. Iron channels and aquaporins on astrocytes can be interrupted by extracellular environmental changes, such as glutamate (Glut), hypoxia and stimulation by cytokines, such as IL-6, IL-1β and TNF, leading to cell swelling and abnormal osmotic forces, followed by cerebral edema and neuronal injury [64, 70, 71]. In addition, astrocytes have proinflammatory potential [64]. Triggered by IL-1β, astrocytes can release VEGF-A and destroy the tight junctions of endothelial cells [72, 73]. Astrocytes can also increase BBB permeability through various mechanisms, including the production of apolipoprotein E (APOE) and inhibition of the cyclophilin A (CYA)–NF-κB–matrix metalloproteinase 9 (MMP9) pathway in pericytes [74].

Pericytes lining along the endothelium play crucial roles in regulating BBB permeability and neuroinflammation [44, 66, 75, 76]. High concentrations of IFN-γ, TNF-α and other cytokines transmitted from serum to the CSF can cause pericyte stress. These cytokines stimulate pericytes to secrete additional inflammatory factors to further activate endothelial cells as positive feedback [39]. The incubation of primary human brain vascular pericytes with IFN-γ and TNF-α can induce large amounts of IL-6 and VEGF secretion [39]. IFN-γ can also inhibit the platelet-derived growth factor receptor β (PDGFRβ) signaling pathway, which is crucial for the proliferation and migration of pericytes, inducing pericyte stress and disrupting their regulatory role in the BBB [77, 78]. Furthermore, a recent study suggested that the on-target, off-tumor effect of CD19 CAR T-cells is also responsible for the breakdown of the BBB. Through single-cell RNA sequencing analysis (scRNA-seq), Parker et al. [79] found that CD19 is expressed on mural cells, including pericytes and vascular smooth muscle cells. These CD19-positive cells can be recognized by CAR T-cells, resulting in pericyte depletion and further BBB disruption. This phenomenon could also explain the higher incidence of ICANS in CD19 CAR T-cell therapy compared with other targets, such as CD22 and CD30 [80–83].

### Inflammatory cellular infiltrates and neuronal dysfunction

Increased BBB permeability allows plasma leakage into the brain parenchyma as confirmed by imaging and histopathologic examinations of patients with CAR T-cell-associated cerebral edema [39, 67, 84–86]. In addition, immune cells and cytokines can penetrate the brain parenchyma and trigger inflammatory reactions in the CNS, thereby inducing neuronal injury and dysfunction. Accumulating evidence sheds light on the contribution of myeloid cells to the pathophysiology of ICANS. Monocytes and macrophages are likely to be recruited to the CNS and produce IL-1β and IL-6, which are key factors in ICANS [20, 87]. Coincidentally, Deng et al. [88] used scRNA-seq to identify rare but significant ICANS-associated cells (IACs), possibly belonging to the myeloid lineage. Moreover, it was demonstrated that GM-CSF, which is crucial for myeloid cell proliferation and activation, was the factor most significantly associated with ICANS in the ZUMA-1 clinical trial [89]. GM-CSF was also highly increased in the CSF of nonhuman primate ICANS models [90]. The increase in GM-CSF may be driven by the diffusion of increased serum GM-CSF or be produced by CNS-infiltrating T cells or activated endothelium. Consequently, GM-CSF promotes the production of IFN-γ-inducible protein 10 (IP-10), MCP-1, and CC chemokine ligand 1 (CXCL1) and attracts myeloid cells to infiltrate the brain [25, 45, 91]. GM-CSF also plays a role in activating microglia [92]. Microglia, which are brain-resident macrophages, can polarize into a proinflammatory phenotype after exposure to GM-CSF, IL-6, and IFN-γ and amplify the inflammatory cascade [93, 94]. Clinically, microglial activation has also been observed in ICANS induced by CAR T-cells [39, 45, 87, 95]. It is assumed that the inflammatory cytokines in the brain parenchyma, including both those produced locally and those traveling from the bloodstream, cause neuron dysfunction and a series of neuropsychiatric symptoms. In preclinical studies, IL-1β and TNF could change neuronal excitability, inducing delirium and other neuropsychiatric symptoms [96, 97]. Other cytokines, such as IL-6 and IL-8, may also be implicated in neural injury [98].

Moreover, myeloid cells are likely to mediate excitotoxicity in ICANS by producing endogenous excitatory agonists [45]. Stimulated by IFN-α2, IFN-γ, and TNF-α, microglia and macrophages can produce the N-methyl-d-aspartate (NMDA) receptor agonists quinolinic acid (QA) and excitatory neurotransmitter Glut, causing seizures and other excitatory symptoms [99, 100]. In addition, QA can promote the secretion of Glut [101], activate astrocytes to secrete numerous cytokines, such as TNF-α, IL-6, and MCP-1, and change the cohesion of the BBB, representing a feed-forward mechanism exacerbating brain dysfunction [101, 102].

Other functions of microglia include synaptic pruning and scavenging damaged cellular debris to maintain hemostasis of the brain and normal cognitive function [103–105]. Abnormalities in these functions contribute to cognitive disorders, which are the main pathophysiology of neurodegenerative diseases and aging and may be implicated in ICANS. Microglia may be depleted through off-tumor effects since low levels of CD19 were detected in human microglia by scRNA-seq and another single-cell transcriptomics database [79, 106]. Moreover, microglia were lost in a murine model treated with CD19 CAR T-cells, which was possibly associated with CD19 targeting [106]. CD22 is also expressed in microglia in the human brain [107], whereas there is no evidence indicating that the severity of ICANS is increased in CD22 CAR T-cell therapy. In summary, the possible on-target off-tumor mechanism of cognitive disorder induced by microglial injury in the ICANS remains to be clarified in further studies.

In addition to the infiltration of myeloid cells and resident microglial activation, T cells and CAR T-cells can also enter the CNS [30, 39, 45, 66, 88, 90]. It was assumed that the cytokines secreted by infiltrated CAR T-cells in the brain constitute another crucial factor causing ICANS [30, 108]. However, CAR T-cells can also be found in CSF from patients without ICANS [45, 66], suggesting that they may play a less important role in neurotoxicity.

## Prevention and management of toxicities

### Optimization of the infusion dose

Because the infusion dose of CAR T-cells and the disease burden are strongly associated with CRS and ICANS [44], Turtle et al. [84] proposed low-dose infusions for patients with high tumor burden, which achieved a high complete remission (CR) rate with no CRS and less ICANS. However, there was a high relapse rate in this study, raising the concern that a reduction in CAR T-cells could impair the long-term prognosis [84]. Recently, split dosing, also called fractionated dosing, has been recommended. This approach delivers CAR T-cells several times in the form of dose escalation, and subsequent infusions can be stopped if early clinical CRS is found [109]. Individualized dose modifications are considered superior to single-dose infusion since they can achieve a balance between the efficacy and safety of CAR T-cell therapy. In the trial reported by Frey et al. [109], there were three schemes of CAR T-cell infusion as follows: high-dose single-infusion (HDS; 5 × 10^8^ CAR T-cells; *n* = 6), low-dose single or fractionated infusion (LD; 5 × 10^7^ CAR T-cells; *n* = 9) and high-dose fractionated infusion (HDF; 5 × 10^8^ CAR T-cells; *n* = 20). In the HDF group, CAR T-cells were planned to be infused within 3 days by split dosing (Day 1, 10%; Day 2, 30%; and Day 3, 60%); however, 9 patients had a high tumor burden; among these patients, 3 received Day 1 only, 3 received Day 1 and Day 2, and the other 3 received all 3 doses. Eighteen patients in the HDF group achieved CR (18/20, 90%), and only 1 patient developed grade 4 CRS (1/20, 5%). In contrast, in the HDS group, only 3 patients achieved CR (3/6, 50%), while the other 3 patients died from CRS and concurrent infections (3/6, 50%). In the LD group, 3 patients (3/9; 33%) achieved CR, and 2 patients experienced CRS > grade 4 (2/9, 22%). The 2-year survival rate was also improved in the HDF group (73% in the HDF group, 22% in the LD group, and 17% in the HDS group). The fractionated dosing scheme was also applied in another clinical trial [110]. The rates of severe CRS and ICANS were decreased in the fractionated infusion cohort, while no remarkable differences in the 1-year progression-free survival (PFS) or 1-year overall survival (OS) were found. Collectively, these data preliminarily demonstrate that split dosing and individualized modification represent promising strategies to alleviate CRS and ICANS. It is more practical for clinicians to adapt infusion dosing than explore drugs or novel CAR T-cell products. More studies are required to validate whether this strategy dampens the efficacy and long-term outcome of CAR T-cell therapy and optimize the specific timing and dosing of infusion.

### Optimization of the CAR structure

The basic components of CAR usually include an antibody-derived single chain variable fragment (scFv), hinge domain (HD), transmembrane domain (TMD), and intracellular domain, which consists of one or more costimulatory domains and a CD3-zeta (CD3ζ) domain. These components all contribute to the transmission of signals for CAR T-cell activation, and optimizing the structure for a safer CAR is a feasible strategy.

Recent studies have indicated that modifying the affinity of scFv for tumor antigens has an impact on toxicities. Ghorashian et al. [111] designed a novel CD19 CAR (CAT) with a lower affinity but more robust cytotoxicity than FMC63, which showed a high CR rate (12/14, 86%) with a low toxicity rate (no severe CRS; 1 severe ICANS) in the treatment of R/R pediatric B-ALL. In addition, the replacement of murine-derived FMC63 with humanized scFv [112] or the addition of an anti-IL-6 antibody to the scFv sequences to neutralize macrophage-derived IL-6 [113] are regarded as possible strategies to mitigate CRS.

The origin, length and flexibility of the HD and TMD also have effects on CAR function and the occurrence of CRS [114–116]. CD8α-HD/TMD and CD28-HD/TMD are most frequently applied in clinical practice, and CD8α-HD/TMD CARs present a lower ICANS incidence [45, 87, 117, 118]. Additionally, including humanized scFv and CD8α-HD/TMD in one CAR molecule may exert synergistic effects on reducing toxicities [119]. In addition to the HD/TMD types, changes in length may impact toxicities. A CD19 CAR T-cell product containing a longer CD8α-HD/TMD was found to be associated with lower cytokine production, inducing no severe CRS or ICANS in 25 patients with refractory B cell lymphoma [116].

Several studies have indicated that CAR T-cells with 4-1BB costimulatory domains are associated with less toxicity than CAR T-cells with CD28 costimulatory domains [45, 120, 121]. A possible explanation for this finding is that CAR T-cells with 4-1BB costimulation have a mild but persistent killing effect mediated by the THEMIS-SHP1 complex, which can counteract the phosphorylation of the CD3ζ domain and consequently downregulate the expression of IFN-γ and other cytokines [122]. In comparison, CD28 CAR T-cells with no THEMIS-SHP1 complex have more rapid and intense phosphoprotein signaling, inducing large amounts of cytokine production and powerful, rapid, but transient killing effects [122]. Based on this mechanism, Sun et al. [122] added the small molecule AP21967 to a modified SHP1 on the CD28 domain to reduce cytokine release and ameliorate CRS without compromising the antitumor effects. However, a retrospective analysis showed that the incidences of CRS and ICANS are likely to be more related to HD and TMD than the costimulatory domain [123]. The role of the costimulatory domain is still unclear and remains to be clarified in further research.

Usually, CD3ζ with 3 immunoreceptor tyrosine-based activation motif (ITAM) domains is contained in the intracellular domain of the CAR and transmits activating signals through ζ-associated protein of 70 kDa (ZAP70) [124]. Reducing the number of ITAMs on CD3ζ or using other CD3 subunits with one ITAM, such as CD3ε, may be effective in inhibiting excessive CAR T-cell activation and decreasing CRS [125–128]. Other optimization strategies include knocking down genes of key cytokines in CAR T-cells that contribute to toxicities, such as GM-CSF [25, 91] and IL-6 [32, 129].

### Elimination switches

Adding switches on CAR T-cells to regulate the inactivation of CAR signaling is being actively explored to eliminate toxicities. Most switches are controlled by small-molecule agents and can be divided into reversible and irreversible switches. The former can reactivate CAR T-cells when the inducers are removed, while irreversible switches induce permanent CAR T-cell damage.

**Fig. 3:**
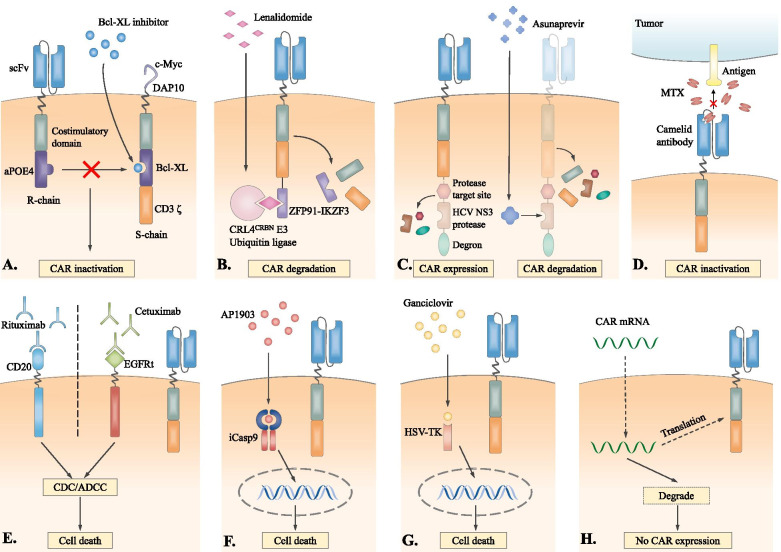
Elimination switches. Reversible switches **A.** The STOP CAR is a dimer of two functional chains that can be disrupted by a Bcl-XL inhibitor. **B.** Lenalidomide induces CRL4^CRBN^ E3 ubiquitin ligase-mediated ubiquitination and degradation of the hybrid zinc finger degron ZFP91-IKZF3-incorporated CAR. **C.** Asunaprevir binds to HCV NS3 protease in SWIFF-CAR and inhibits the degradation of the protease/degron complex. Therefore, the whole CAR would be degraded by degron. **D.** The CAR contains a conditional scFv based on the camelid antibody. MTX binds to the scFv and induces scFv conformational changes, therefore inhibiting CAR from recognizing TAA. Irreversible switches **E.** Upon the administration of monoclonal antibodies, CAR T-cells expressing CD20 or EGFRt can be irreversibly eliminated through the CDC or ADCC effect. **F.** The administration of AP1903 induces the dimerization of iCasp9, which triggers downstream apoptotic cascades, resulting in CAR T-cell death. **G.** When ganciclovir is administered, HSV-TK phosphorylates ganciclovir to form the toxic ganciclovir-triphosphate compound, leading to the inhibition of DNA synthesis and CAR T-cell death. **H.** T-cells transfected with mRNA transiently encode CAR, the expression of which can be downregulated with mRNA degradation

Reversible switches can be achieved through the chemically disruptable heterodimerization of functional chains. Giordano-Attianese et al. [130] designed a STOP CAR (Fig. 3A) that contains the following 2 chains: recognition (R) chain and signaling (S) chain. The extracellular domain of the S chain consists of a c-Myc-tag and DAP10, and DAP10 maintains the expression stability of the S chain. The two chains are spontaneously dimerized through a computationally designed protein pair, apolipoprotein E4 (apoE4) and B cell lymphoma-extra large (Bcl-XL). ApoE4 is expressed at the end of the R chain, and Bcl-XL is located between the CD28 costimulatory domain and the CD3 ζ domain of the S chain. The specific disruption between apoE4 and Bcl-XL is triggered by the Bcl-XL inhibitor A1155463, consequently interfering with CAR signaling and transiently inactivating CAR T-cells. Another off-switch design is combined with proteolysis-targeting chimera (PROTAC) technology and triggered by lenalidomide (Fig. 3B) [131]. The hybrid zinc finger degron tag ZFP91-IKZF3 is added at the C-terminus of the CAR. The presence of lenalidomide can induce CRL4^CRBN^ E3-mediated ubiquitylation and proteasomal degradation of CAR, while CAR signaling can be restored by decreasing the lenalidomide levels. Although the two forms of switches mentioned above have not been used in clinical settings, they have good clinical suitability since these switches originate from nonimmunogenic human sequences, and the small molecule agents are FDA-approved. Additionally, CAR molecules can be incorporated with cleavable degradation moieties (degrons) called switch-off CARs (SWIFF-CARs) (Fig. 3C) [132]. SWIFF-CARs are controlled by hepatitis C virus (HCV) NS3 protease, and the administration of asunaprevir can inhibit the cleavage activity of HCV NS3 protease, preventing the degradation of protease/degron complex from CAR. As a result, the degron induces the rapid degradation of CAR. However, the strategies mentioned above all require the addition of a switch gene, which is challenging for the vector virus loaded with a CAR gene since the viral payload capacity is limited. Therefore, Park et al. generated a conditional scFv based on a camelid antibody, which is capable of recognizing both tumor-associated antigen (TAA) and methotrexate (MTX) **(**Fig. 3D) [133]. Upon binding MTX, the conformation of MTX-based scFv changes to disrupt TAA binding, resulting in CAR T-cell inactivation [133]; when MTX is removed, CAR T-cells are reactivated.

Alternatively, irreversible switches also represent a strategy to eliminate the activities and toxicities of CAR T-cells. It can be achieved through complement-dependent cytotoxicity (CDC) and antibody-dependent cell-mediated cytotoxicity (ADCC). CAR T-cells engineered with surface antigens can be neutralized by FDA-approved therapeutic antibodies, such as rituximab (targeting CD20) [134] and cetuximab (targeting truncated epidermal growth factor receptor, EGFRt) [135], and cause irreversible clearance **(**Fig. 3E). However, this strategy takes effect slowly and, therefore, may be unsuitable for patients with severe cytokine-mediated toxicities requiring urgent treatment. Another feasible solution is to express inducible caspase 9 (iCasp9) in CAR T-cells **(**Fig. 3F). iCasp9 is an apoptosis-triggering fusion protein, and rimiducid (AP1903) can induce the dimerization of iCasp9 and activate downstream caspase, eliminating the engineered CAR T-cells within 30 min [136, 137]. This strategy induced dramatic and immediate CAR T-cell elimination in a 26-year-old female, effectively alleviating the ICANS symptoms and improving the ICANS grades (from 3 to 1) and the Immune Effector Cell-Associated Encephalopathy (ICE) score (from 0 to 7) within 12 h [138]. The efficacy of iCasp9 CAR T-cells in CD19 B cell malignancies is still being evaluated in clinical trials (NCT03016377 and NCT03696784). Herpes simplex virus thymidine kinase (HSV-tk)/ganciclovir is another safety switch that inhibits DNA synthesis through death-inducing signaling cascades and, therefore, induces CAR T-cell death **(**Fig. 3G**)**. However, its clinical applications are likely to be limited by its immunogenicity and slow induction of cell death [139, 140]. In addition, transfection with mRNA can construct a ‘biodegradable’ product with the transient expression of CAR molecules. CAR is downregulated along with the degradation of mRNA [141], reducing the risk of supercytokine production from the long-term overactivation of CAR T-cells **(**Fig. 3H**)**. However, to achieve remission, multiple infusions of such CAR T-cells are required, which may increase the risk of phylaxis [142].

In summary, the off-switch is a promising strategy to eliminate toxicities, especially reversible switches, which avoid the permanent elimination of costly and robust CAR T-cells. More studies are expected to explore the on/off kinetics of such switches and confirm their immediate reactivity in vivo before translation into the clinic. Setting a specific timing for clinicians to turn off the switch is also of great importance. Moreover, safety issues associated with the small-molecule controller should be considered, and using approved agents as controllers may be better choices. In the future, clinical validations of the antitumor effects and toxicity-management ability of these novel products are needed.

### Anticytokine agents

#### Targeting IL-6

Tocilizumab is a humanized IgG1 monoclonal antibody that blocks both membrane-bound and soluble IL-6 receptors, inhibiting IL-6, the key mediator in the pathogenesis of CRS. As the only FDA-approved and first-line agent for severe or life-threatening CRS [143] **(**Table 3**)**, tocilizumab effectively induces the rapid reversal of symptoms and normalizes laboratory markers in most patients [31, 144]. Although it was assumed that inhibiting the downstream pathways of IL-6 may disrupt the cytokine environment necessary for maintaining CAR T-cell potency, clinical experience has shown that tocilizumab does not dampen their expansion, persistence and antitumor efficacy [89, 145].

**Table 3:** Summary of potential anticytokine agents for CAR-T associated CRS and/or ICANS

Agent	Target	Application	Mechanism	Stage and clinical trial
Tocilizumab	IL-6	CRS	Blocking IL-6R, inhibiting IL-6, the key cytokine of the CRS	FDA-approved first-line agent for severe CRS [143]
Siltuximab	IL-6	CRS ICANS	Blocking IL-6	Clinical trial [152]
Corticosteroids	NA	CRS ICANS	Non-specific anti-inflammatory effects to suppress immune cells	First-line agent for severe and isolated ICANS [156]
Anakinra	IL-1	CRS ICANS	Blocking IL-1, an important cytokine in CRS and ICANS	Ongoing trials: NCT04148430, NCT04150913, NCT04205838, NCT04432506, NCT04359784, NCT03430011, NCT04227275
Lenzilumab	GM-CSF	CRS ICANS	Blocking GM-CSF and inhibiting myeloid cells and T cells entering CNS	Ongoing trial: NCT04314843
Ruxolitinib	JAK1/2	CRS	Broadly inhibiting JAK-STAT pathways, the downstream of multiple cytokines	Clinical trial [175]
Itacitinib	JAK1	CRS	Selectively inhibiting the JAK-STAT pathways	Ongoing trial: NCT04071366
Dasatinib	TK	CRS	Blocking the adenosine triphosphate binding sites of LCK, reversibly inhibiting the activation of CAR T-cells	Ongoing trial: NCT04603872
Ibrutinib	ITK	CRS ICANS	Inhibiting the ITK-induced cytokine release of T cells, monocytes and tumor cells	Ongoing trials: NCT04234061, NCT03331198, NCT03310619, NCT04640909, NCT03570892
Metyrosine	Catecholamine	CRS	Blocking tyrosine hydroxylase to inhibit the synthesis of catecholamine	Preclinical [28]
ANP	Catecholamine	CRS	Inhibiting cytokine secretion	Preclinical [28]
Etanercept	TNF-α	CRS	Blocking TNF-α, an important cytokine in CRS	Clinical trials [29, 192, 193, 225]
Adalimumab	TNF-α	CRS ICANS	Blocking TNF-α	Preclinical, administered with anti-IL-1β antibody [194]
Extracorporeal cytokine removal Plasma exchange Hemofiltration	NA	CRS ICANS	Removal of pro-inflammatory mediators from the blood	Ongoing trial: NCT04048434
TO-207	mRNA 3′-end	CRS	An mRNA 3′-end processing antagonist, inhibiting the secretion of multiple cytokines	Preclinical [196]
THZ1	CDK7	CRS	Suppressing a set of inflammatory genes, mainly STAT and IL-1	Preclinical [197]

*CAR-T* Chimeric antigen receptor T cell, *CRS* Cytokine release syndrome, *ICANS* Immune effector cell associated neurotoxicity syndrome, *IL* Interleukin, *FDA* US Food and Drug Administration, *NA* Not applicable, *GM-CSF* Granulocyte-macrophage colony-stimulating factor, *JAK* Janus kinase, *STAT* Signal transducer and activator of transcription, *TK* Tyrosine kinase, *LCK* Lymphocyte-specific protein tyrosine kinase, *ITK* IL-2-induced tyrosine kinase, *TNF-α* Tumor necrosis factor-α, *CDK7* Cyclin-dependent kinase 7

Tocilizumab is usually applied in cases of severe CRS, while mild CRS can be resolved through supportive care alone [146, 147]. Consensus regarding the specific timing for tocilizumab administration has not been reached and is an active field of investigation. The latest research shows that early intervention (EI) for CRS may be a better option by administering tocilizumab within a short time after CAR T-cell infusion or at the onset of mild CRS symptoms [145, 148]. Gardner et al. [145] found that the EI strategy with tocilizumab and/or corticosteroids could reduce the incidence of severe CRS in ALL patients receiving CD19 CAR T-cells without dampening the antitumor efficacy. Another clinical trial also demonstrated that the EI strategy was effective for high-tumor-burden B-ALL [149]. More aggressively, in some studies, tocilizumab was even administered before the infusion of CAR T-cells and led to a decrease in subsequent CRS [150].

However, tocilizumab has shown limited efficacy in most ICANS cases [39, 45]. Early intervention with tocilizumab may even aggravate ICANS. In a safety expansion trial of ZUMA-1, prophylactic tocilizumab was administered to NHL patients 2 days after the CAR T-cell infusion, and an increase in severe ICANS was observed compared with the original research in which tocilizumab was administered after the occurrence of CRS; in addition, one death occurred due to cerebral edema [89, 148]. The reason for ICANS aggravation is likely to be the increased BBB permeability to IL-6 and poor BBB penetration of tocilizumab. After the extensive blockade of IL-6R in the peripheral circulation, large amounts of IL-6 enter the BBB through passive diffusion and trigger an inflammatory response in the CNS, which cannot be neutralized by tocilizumab [151].

Siltuximab may be an alternative agent to tocilizumab. By binding IL-6, siltuximab removes IL-6 from the circulation [33], thus reducing IL-6 entry into the CNS and alleviating ICANS. Shah et al. successfully resolved CRS and ICANS with either tocilizumab, corticosteroids, and/or siltuximab in 5 patients [152], preliminarily showing the efficacy of siltuximab. However, to date, solid clinical evidence is lacking, and more research is needed to verify the therapeutic effects of siltuximab.

#### Corticosteroids

Corticosteroids have been shown to effectively resolve the symptoms of CRS and ICANS after CAR T-cell infusion by exerting powerful nonspecific anti-inflammatory effects [31, 153, 154]. Corticosteroids are the first-line therapy for isolated ICANS [155, 156] **(**Table 3**)**, while for CRS, corticosteroids are usually reserved for life-threatening (grade 3 or 4) or tocilizumab-resistant CRS [151, 155, 157]. Dexamethasone is the most commonly used among corticosteroids because it shows good CNS penetration and can improve the integrity of the BBB [48, 151, 158].

A concern is that corticosteroids may compromise the antitumor efficacy by dampening T-cell function, expansion, and persistence and even inducing T cell apoptosis [153, 159], and it was found that using corticosteroids reduced the OS rate of patients receiving CAR T-cell therapy in a clinical trial [160]. However, increasing evidence indicates that rational dosage regimens of corticosteroids could maintain the intact efficacy of CAR T-cell therapy. It is likely that the short-term use of corticosteroids is practicable [161]. Moreover, the earlier administration of corticosteroids is under investigation [145, 162–164]. The ZUMA-3 trial administered corticosteroids at grade 2 ICANS after CD19 CAR T-cell therapy [162]. Compared with administering corticosteroids at grade 3 ICANS, there were significant reductions in the incidence rates of severe ICANS (11% vs 64%) and peak levels of cytokines and other proinflammatory markers in the early intervention group; CAR T-cell expansion was similar between the two groups [162]. A more aggressive measure may also be feasible. A safety management cohort of ZUMA-1 adopted the prophylactic administration of corticosteroids during the first 3 days after the CAR T-cell infusion and early corticosteroid and/or tocilizumab intervention for grade 1 CRS or ICANS, which resulted in no severe CRS and low rates of severe ICANS (13%) without detrimental effects on the efficacy of CAR T-cells [164]. In clinical practice, further explorations are needed to optimize corticosteroid regimens, including their dosage, timing and duration.

#### Targeting IL-1

In addition to IL-6 blockade and corticosteroids, other anticytokine candidates are being actively explored and are summarized in Table 3**.** One potential strategy is targeting IL-1. IL-1β, which is released earlier than IL-6 and promotes IL-6 production, is another key cytokine involved in CRS and ICANS [20, 165]. Anakinra, an FDA-approved recombinant IL-1 receptor antagonist for neonatal onset multisystem inflammatory disease (NOMID) and rheumatoid arthritis (RA), has been proven to successfully resolve CRS in mouse models without affecting the expansion of CAR T-cells [21]. In addition, notably, anakinra can penetrate the BBB and target IL-1β in the CNS [166], ameliorating ICANS symptoms and the histopathological signs of meningeal inflammation in a mouse model, which cannot be resolved by tocilizumab [20]. A clinical trial confirmed that anakinra is effective in the treatment of hemophagocytic lymphohistiocytosis (HLH) after CD22 CAR T-cell therapy in ALL patients [81]. Strati et al. applied anakinra in 6 patients with severe ICANS refractory to tocilizumab and dexamethasone, and 4 of the 6 patients showed improvement in symptoms [167]. It is hypothesized that a high disease burden and early progression limit the efficacy of anakinra since it was used as the last remedy for toxicities in this trial, and the preemptive administration of anakinra may result in better outcomes. More relevant clinical trials are ongoing (NCT04148430, NCT04150913, NCT04205838, and NCT04432506).

#### Targeting GM-CSF

GM-CSF is also an important cytokine associated with CAR T-cell-related adverse events, especially ICANS [89, 91]. Lenzilumab, a monoclonal antibody against GM-CSF, can resolve ICANS and CRS induced by CAR T-cells in mouse models by inhibiting myeloid cells and T cells entering the CNS [91]. Intriguingly, instead of dampening CAR T-cell expansion, lenzilumab even enhanced its antitumor effect [91]. The ZUMA-19 trial (NCT04314843) preliminarily showed the efficacy and safety of lenzilumab and Axi-Cel (an FDA-approved CD19 CAR T-cell product) in treating patients with relapsed or refractory diffuse large B cell lymphoma (DLBCL). It was observed that lenzilumab could induce a lower risk of severe CRS and ICANS and reduce inflammatory markers, such as IL-6, CRP, ferritin, MCP-1, IL-8, and IP-10 [168].

#### Targeting the JAK signaling pathway

The JAK family consists of JAK1, JAK2, JAK3, and tyrosine kinase 2 (TYK2), which are bound to the intracellular domains of type I and type II cytokine receptors, including interleukins, IFNs, and colony-stimulating factors, and regulate gene transcription by phosphorylating STAT [169]. Different JAK subunits selectively bind different cytokine receptors, while compared with other JAKs, JAK1 plays a broader role [170]. In terms of IL-6, its receptor binds JAK1, JAK2 and TYK2, and JAK1 is the dominant kinase [171, 172].

Some researchers are exploring JAK inhibitors for CRS treatment. Ruxolitinib, a JAK1/2 inhibitor approved for the treatment of myeloproliferative neoplasms (MPN) and steroid-refractory acute graft-versus-host disease (GVHD) [173, 174], showed feasibility in preventing severe CRS refractory to steroid therapy in four R/R B-ALL patients receiving CD19 or CD22 CAR T-cell therapy [175]. Grade 3 CRS induced by CD7 CAR T-cells was also resolved by ruxolitinib-based therapy in 2 T-ALL patients [176]. In addition, itacitinib, which is a selective JAK1 inhibitor developed for the treatment of graft-versus-host disease, may resolve CRS with a lower risk of immunosuppression [177]. A phase 2 trial evaluating itacitinib for the prevention of CRS is ongoing (NCT04071366).

#### Tyrosine kinase inhibitor

Dasatinib, an FDA-approved tyrosine kinase inhibitor (TKI), blocks the adenosine triphosphate binding sites of lymphocyte-specific protein tyrosine kinase (LCK) and inhibits the phosphorylation of CD3ζ and ZAP70 [178, 179], effectively depressing the activation of CAR T-cells and T cells. Mestermann et al. demonstrated the on/off switch effect of dasatinib [180]. Dasatinib can transform CAR T-cells into a functional off state both in vitro and in a mouse model, reversibly inhibiting the proliferation and cytotoxicity of CAR T-cells. Upon the removal of dasatinib, CAR T-cell functions can be quickly restored without compromising the overall efficacy. Moreover, during the pause phase, CAR T-cells can undergo epigenetic remodeling to reverse exhaustion, thereby enhancing the cell fitness and antitumor potency [181]. Since dasatinib directly interferes with the CAR T-cell signaling pathway, inhibiting CAR T-cells more rapidly and completely than dexamethasone, which regulates transcription, may be suitable for the emergency treatment of CRS [180].

Ibrutinib is a Bruton’s tyrosine kinase (BTK) inhibitor approved by the FDA for the treatment of chronic lymphocytic leukemia (CLL). Ibrutinib can also inhibit the IL-2-induced tyrosine kinase (ITK) pathway, regulating the cytokine release of various immune cells, such as T cells, monocytes and tumor cells [182–184]. Several trials have demonstrated that the concurrent use of ibrutinib and CAR T-cells for CLL can exert synergistic antitumor efficacy [185, 186] while ameliorating CRS [185].

#### Other rational therapies

With an evolving understanding of the mechanisms of CRS and ICANS, more potential therapeutic targets are emerging. Transforming pyroptosis into apoptosis and disrupting the onset of CRS by inhibiting the production of gasdermins, inflammasomes, caspases or DAMPs or regulating the killing pathway of the CAR appear promising [187–189]. Based on the role of catecholamines in CRS, experiments in a mouse model have shown that the early use of metyrosine (MTR) or atrial natriuretic peptide (ANP) can prevent CRS by reducing the production of catecholamines and various cytokines without affecting the efficacy of CAR T-cells [28]. It was previously emphasized that the activation of endothelial cells plays an important role in CAR T-cell toxicities, especially ICANS. Strategies, such as rescuing the disorder of the Ang-Tie2 axis by Ang I or vascular endothelial protein tyrosine phosphatase (VE-PTP)-dependent restoration [59, 190] and using defibrotide to regulate endothelial cell-leukocyte interactions [191], may be promising for reversing hemodynamic disorders and maintaining the stability of the BBB as a result abrogating ICANS.

Blocking other important cytokines in the development of CRS and ICANS to interrupt the inflammatory cascade is also a potential strategy. Some centers have attempted to use etanercept to block TNF-α, and its feasibility has been preliminarily confirmed [29, 192, 193]. Moreover, an in vitro experiment demonstrated that the use of adalimumab, a TNF-α antibody, combined with an anti-IL-1β antibody could exert a synergistic effect on preventing the activation of endothelial cells [194]. Emapalumab, which blocks IFN-γ in the treatment of HLH [195], may also inhibit CAR T-cell-associated toxicities but poses a significant risk to CAR T-cell efficacy [6]. By preventing mRNA maturation, TO-207 can abrogate the monocyte production of multiple cytokines, such as TNF-α, IL-6, IL-1β, MCP-1, IL-18, IL-8, and GM-CSF, without dampening the cytotoxicity of CAR T-cells [196]. Pretreatment with THZ1 to block cyclin-dependent kinase 7 (CDK7) may also be an option. THZ1 can suppress inflammatory genes at the transcriptional level, particularly inhibiting STAT1 and IL-1, therefore, alleviate CRS without impairing the antitumor efficacy [197]. In addition, nonspecific cytokine filtering through extracorporeal cytokine removal, plasma exchange, or hemofiltration is also effective [198–200].

## Conclusion and future perspectives

The toxicities of CAR T-cell therapy, especially CRS and ICANS, impede the broader adoption of this robust antitumor agent. Studies investigating the mechanisms of CRS have made great breakthroughs. The key involvement of pyroptosis is highlighted, and the role of macrophages as the main source of important cytokines in CRS has been confirmed. Regarding ICANS, some crucial links between the main cellular and molecular mediators are still elusive. It is reasonable to speculate that the mechanisms of common reversible neurocognitive disorders differ from those of lethal events, and further studies are warranted to explain the wide spectrum of clinical manifestations. Animal models play critical roles in both further understanding the detailed mechanisms of toxicities and exploring promising strategies. Rapid progress is underway in the optimization of the modeling, including humanized mice and primate models, in an effort to narrow the gap with clinical settings. In addition, advances in sequencing and omics techniques provide new insight into mechanistic studies. Such techniques can help identify certain cell populations and components that cannot be identified by traditional technologies. The development of multidimensional parameter analyses, combining preclinical and clinical evidence and collaborating phenotypic and functional information is expected to provide more comprehensive insight into the underlying mechanisms.

For the prevention and management of toxicities, strategies have been proposed from different perspectives. Optimizing the dosing schemes is a practical clinical strategy, and laboratory strategies include developing less toxic CAR structures and reversible off-switches, which may radically improve the safety of CAR T-cell therapy. However, more research is still required before these strategies can be broadly available in clinical settings. Currently, the most popular intervention is pharmacotherapy. Tocilizumab, as the staple of CRS treatment, and corticosteroids, another common agent for CRS and ICANS, are being explored for preemptive toxicity mitigation and gradually showing their superiority. More efforts are expected to determine the optimal dosing and timing for prophylactic use. Notably, ideal targeted interventions for ICANS are lacking, and recent studies are shedding light on other specific immune modulators, such as inhibitors of IL-1 and GM-CSF. Since mechanisms are being gradually unveiled, it is hopeful that they will provide more insight for novel intervention strategies. For instance, targeting pyroptosis is a future direction to abolish the onset of CRS. In general, ideal solutions for toxicity are effective without a negative impact on the effectiveness of CAR T-cells and are easy to implement in most medical centers in an effort to promote the widespread use of a safer product in real-world practice.

CAR T-cell therapy is also broadening its field to various cancers and nonmalignant diseases, including infections, autoimmune diseases, transplantation, cardiac fibrosis and senescence-associated pathologies [201–204]. The manifestations, severities, and outcomes of such toxicities are likely to differ mainly in terms of the primary diseases, distinctive targeting cells and different categories of T cells adopted for CAR T-cell engineering. It is hoped that the mechanisms summarized in this article could facilitate an understanding of the toxicities of different indications. Simultaneously, deeper investigation of their unique features and underlying mechanisms is of great importance, which can aid the accurate recognition of toxicities and the development of rational management strategies, enabling the application of safer CAR T-cell therapy in a wider range of fields and providing greater benefits to more patients.

## Data Availability

Not applicable.

## Abbreviations

CAR: Chimeric antigen receptor
FDA: US Food and Drug Administration
CRS: Cytokine release syndrome
ICANS: Immune effector cell associated neurotoxicity syndrome
ALL: Acute lymphocyte leukemia
BCMA: B cell maturation antigen
R/R MM: Relapse or refractory multiple myeloma
MHC: Major histocompatibility complex
ASTCT: American Society for Transplantation and Cellular Therapy
GM-CSF: Granulocyte-macrophage colony-stimulating factor
GZMB: Granzyme B
GZMA: Granzyme A
GSDME: Gasdermin E
GSDMB: Gasdermin B
CTLs: Cytotoxic T lymphocytes
DAMPs: Damage-associated molecular patterns
HMGB1: High-mobility group box 1
ATP: Adenosine 5′-triphosphate
dsDNA: Double-stranded DNA
TLR: Toll-like receptor
MyD88: Myeloid differentiation primary-response 88
TRIF: TIR-domain-containing adaptor inducing IFNβ
MAPKs: Mitogen activated protein kinases
IKK: IκB kinase
AP-1: Activator protein 1
NF-κB: Nuclear factor κB
sIL-6R: Soluble IL-6R
NLRP3: NOD-, LRR-, and pyrin domain-containing protein 3
ASC: Apoptosis-associated speck-like protein
AIM2: Absent in melanoma 2
CD40L: CD40 ligand
iNOS: Inducible nitric oxide synthase
AR: α1-adrenergic receptors
MIP-1α: Macrophage inflammatory protein-1α
JAK: Janus kinase
STAT3: Signal transducer and activator of transcription 3
mIL-6R: Membrane-bound IL-6 receptor
VEGF: Vascular endothelial growth factor
MCP-1: Monocyte chemoattractant protein-1
PAI-1: Plasminogen activator inhibitor-1
TF: Tissue factor
L-CRS: Local CRS
NHL: Non-Hodgkin lymphoma
BBB: Brain blood barrier
CNS: Central nerve system
PVS: Perivascular space
EBM: Endothelial basement membrane
PBM: Parenchymal basement membrane
Ang: Angiopoietin
W-P: Weibel-Palade
VE cadherin: Vascular endothelial cadherin
DIC: Disseminated intravascular coagulation
vWF: Von Willebrand factor
ADAMTS13: A disintegrin and metalloproteinase with thrombospondin type 1 repeats member 13
TTP: Thrombotic thrombocytopenic purpura
GFAP: Glial fibrillary acidic protein
Glut: Glutamate
APOE: Apolipoprotein E
CYA: Cyclophilin A
MMP9: Matrix metalloproteinase 9
PDGFRβ: Platelet-derived growth factor receptor β
scRNA-seq: Single-cell RNA sequencing analysis
IACs: ICANS associated cells
IP-10: IFN-γ-inducible protein 10
CXCL1: CC chemokine ligand 1
NMDA: N-methyl-d-aspartate
QA: Quinolinic acid
CR: Complete remission
HDS: High-dose single-infusion
LD: Low-dose single or fractionated infusion
HDF: High-dose fractionated infusion
PFS: Progression-free survival
OS: Overall survival
scFv: Single chain variable fragment
HD: Hinge domain
TMD: Transmembrane domain
ITAM: Immunoreceptor tyrosine based activation motif
ZAP70: ζ-associated protein of 70 kDa
R: Recognition
S: Signaling
apoE4: Apolipoprotein E4
Bcl-XL: B cell lymphoma-extra large
PROTAC: Proteolysis-targeting chimera
SWIFF-CARs: Switch-off CARs
HCV: Hepatitis C virus
TAA: Tumor-associated antigen
MTX: Methotrexate
CDC: Complement dependent cytotoxicity
ADCC: Antibody-dependent cell-mediated cytotoxicity
EGFRt: Truncated epidermal growth factor receptor
iCasp9: Inducible caspase 9
ICE: Immune Effector Cell Encephalopathy
HSV-tk: Herpes simplex virus thymidine kinase
EI: Early intervention
NOMID: Neonatal onset multisystem inflammatory disease
RA: Rheumatoid arthritis
HLH: Hemophagocytic lymphohistiocytosis
DLBCL: Diffuse large B cell lymphoma
TYK2: Tyrosine kinase 2
MPN: Myeloproliferative neoplasms
GVHD: Graft-versus-host disease
TKI: Tyrosine kinase inhibitor
LCK: Lymphocyte-specific protein tyrosine kinase
BTK: Bruton’s tyrosine kinase
CLL: Chronic lymphocytic leukemia
ITK: IL-2-induced tyrosine kinase
MTR: Metyrosine
ANP: Atrial natriuretic peptide
VE-PTP: Vascular endothelial protein tyrosine phosphatase
CDK7: Cyclin-dependent kinase 7
